# Research and Remedies

**Published:** 1912-02-10

**Authors:** 


### RESEARCH AND REMEDIES.

### Salpingostomy.

In a paper read to the Harveian Society and published in the *Lancet*, Dr. F. J. McCann advocates strongly the operation of salpingostomy in the treatment of sterility due to adhesions and other remains of inflammatory troubles of the Fallopian tubes. He insists, in our opinion very properly, upon the folly of indiscriminate curetting in these cases; and points out how futile the operation is when the ostia or the lumina of the tubes are sealed by past gonorrhœal or other salpingitis. He might also add that in these cases the operation, besides being useless, is actually dangerous in stirring up old quiescent troubles. Dr. McCann strongly urges exploratory laparotomy whenever a sterile woman anxious for children is found to have thickened tubes or a uterus even partially fixed by adhesions. He emphasises especially the need for using the finest needles and sutures in performing the salpingostomy, the advantage of performing a ventro-suspensory operation at the same time, and his plan of using the omentum to reinforce the suspending structures. Several cases are quoted showing the benefit which has followed this operative plan of campaign; but it would be instructive also to have a tabular account of the percentage of failures. Necessarily this must be large, since the operation is done too often after other measures have failed, and upon subjects whose pelvic organs are certainly most unfavourably handicapped for conception. The other principal pleas in a very comprehensive survey of the subject are for early operative interference in these patients, and for simple dilatation as opposed to actual curetting in those whose pelvic organs display no demonstrable abnormality of inflammatory origin.

### The Ætiology of Oral Carcinoma.

A very careful discussion of the ætiology of oral carcinoma has been contributed to the *Quarterly Journal of Medicine* by Dr. C. Singer, who has submitted to analysis an enormous number of fully recorded cases. The provisional conclusions towards which he tentatively inclines are as follows:—

Carcinoma beginning in the oral mucous membrane may form a separate clinical entity, in which carcinoma of the œsophagus should perhaps be included, but from which epithelioma of the lip should probably be excluded. The cases in this oral group of cancer do not, as a class, form an average sample of the general population; they appear to differ in the following particulars: (*a*) There is an overwhelming preponderance of males over females among them. (*b*) A large percentage have suffered from syphilis, often of a very severe type. (*c*) Many are stout, heavy, plethoric men of the type well illustrated in actual life by our police and soldiers. They are most often men of exceptionally robust previous health. These facts are emphasised by their social position and occupations. (*d*) That their metabolism is probably not that of the normal population is suggested by the fact that a considerable percentage of them have suffered more or less severely from gout, usually of a typical and easily recognisable form. (*e*) Although apparently healthy, except as regards the local disease and the effects of syphilis and gout, these patients exhibit evidence of renal interstitial change and arterial degeneration; though it is true that such degenerations are apt to declare themselves at a period of life when carcinoma of the mouth is most common. Yet even allowing for this, and allowing for syphilis and gout as antecedent conditions, there still appears to be a further unexplained predominance of vascular and renal change among cases of oral cancer.

The liability to oral carcinoma at various ages differs from that of cancer of other regions in a special manner; this difference may be graphically expressed in death-rate curves which are characteristic of this malady and are typified by the curves for cancer of the tongue. It is suggested that these peculiarities may be in part explained by assuming that the earlier cases are more frequently of syphilitic origin, while the later have other associations, among which gout is to be reckoned. Aneurysm and certain vascular diseases present certain analogies to some types of oral carcimona, especially as regards age distribution, and it seems not unlikely that these analogies may be related to similar ætiological factors. That at least is the opinion of Dr. Singer, whose position of Medical Registrar to the Cancer Hospital gives him many opportunities of observing medical aspects of these malignant cases.

### Secondary Infections in Phthisis.

Of late it has become the fashion to attribute a large part of the symptoms of pulmonary tuberculosis to the effects of superadded secondary infections rather than to the toxins of the bacillus tuberculosis itself. It has even been suggested by some that rises of temperature are proof of secondary infection, which is supposed to be universal and inevitable in all cases of "open" tuberculosis of the lungs. After an investigation conducted for the Local Government Board, Dr. A. C. Inman contests these assertions, and gives his reasons for his belief that they are not absolutely accurate. He finds that in nearly every case of open tuberculosis of the lungs the bacillus tuberculosis is the predominant infecting agent. The blood examinations which he conducted do however show that secondary infections occur; and even in the ambulant afebrile cases such infection cannot be excluded. The temperature chart alone cannot determine the presence or absence of a secondary infection, as some observers have maintained. Lastly, he says that a consideration of the morbid anatomy of advanced tuberculosis and of the uncontrolled, spontaneously occurring auto-inoculations in such cases, precludes the hope of a successful issue from specific treatment directed against the secondary infections; but the situation is more encouraging in the afebrile cases than in the others.

